# MAIRA- real-time taxonomic and functional analysis of long reads on a laptop

**DOI:** 10.1186/s12859-020-03684-2

**Published:** 2020-09-17

**Authors:** Benjamin Albrecht, Caner Bağcı, Daniel H. Huson

**Affiliations:** 1 Department of Computer Science, University of Tübingen, Sand 14, Tübingen, Germany; 2 International Max Planck Research School From Molecules to Organisms, Max Planck Institute for Developmental Biology and Eberhard Karls University Tübingen, Max-Planck-Ring 5, Tübingen, 72076 Germany

**Keywords:** Metagenomics, Microbiome, Long read sequencing, Taxonomic analysis, Functional analysis, Mobile computing, Open source software, Antibiotic resistance, Virulence factors

## Abstract

**Background:**

Advances in mobile sequencing devices and laptop performance make metagenomic sequencing and analysis in the field a technologically feasible prospect. However, metagenomic analysis pipelines are usually designed to run on servers and in the cloud.

**Results:**

MAIRA is a new standalone program for interactive taxonomic and functional analysis of long read metagenomic sequencing data on a laptop, without requiring external resources. The program performs fast, online, genus-level analysis, and on-demand, detailed taxonomic and functional analysis. It uses two levels of frame-shift-aware alignment of DNA reads against protein reference sequences, and then performs detailed analysis using a protein synteny graph.

**Conclusions:**

We envision this software being used by researchers in the field, when access to servers or cloud facilities is difficult, or by individuals that do not routinely access such facilities, such as medical researchers, crop scientists, or teachers.

## Background

The Oxford Nanopore MinION USB sequencing device, the MinIT USB base-calling device and advances in lab-on-a-chip technologies allow sequencing to be taken into field [1]. With ever rising sequencing yield and continuously growing reference databases, the computational analysis of such data is challenging, and much work is being done to address this efficiently, usually on a server or using cloud computing [2, 3]. Modern laptops provide a lot of computational power, main memory and fast access to SSD storage, and thus it should be possible to perform detailed analysis of microbiome sequencing data in the field, on a laptop.

In this paper, we present a new program called MAIRA (mobile analysis of long reads) for analyzing long metagenomic reads on a laptop, without requiring external resources. The program first performs fast genus-level analysis in real time, and then also performs detailed species-level taxonomic and functional analysis. The latter refers to basic analysis of antibiotic resistance potential [4] and virulence factors [5].

We envision this software being used by researchers in the field, when access to servers or cloud facilities is difficult [6], or by individuals that do not routinely use such facilities, such as medical researchers, crop scientists, or teachers, say. The program is able to analyze sequencing data in real-time and thus may be applicable in time-critical applications.

Following the approach established in [7, 8], we base the taxonomic and functional alignment of metagenomics sequencing reads on the alignment of the reads against a protein reference database; here we use all bacterial proteins in RefSeq [9].

There are three main challenges.

1. Current long reads contain many erroneous insertions and deletions, causing frame shifts that disrupt translated alignments and result in very poor performance of methods such as BLASTX [10].
2. The alignment of gigabases of sequencing reads against a reference database containing on the order of one hundred million reference proteins requires substantial computational resources, despite major advances [11, 12].
3. For many applications, such as the assessment of antibiotic resistance or virulence, say, it is crucial to link the associated genes to specific organisms.

Here we describe a new open source program called MAIRA that addresses these three main challenges.

## Implementation

Challenge 1 can be solved using a frame-shift aware alignment tool [13], and MAIRA supports the use of LAST [11], but also contains a new, built-in frame-shift aligner called ELLA (manuscript in preparation). To address Challenge 2, the program performs a fast online genus-level analysis, whereas a more detailed analysis can be computed on-demand, for selected genera of interest. To address Challenge 3, we introduce the concept of a protein synteny graph (manuscript in preparation) whose nodes represent gene families and whose edges reflect synteny.

MAIRA is a standalone computer program for analyzing sequencing reads from a mobile sequencing device or long reads from other sources. MAIRA is written in Java and runs on all three major operating systems. The program is designed to be used interactively, however a command-line mode is also provided.

### Online and on-demand

Initial analysis is performed “online” in the sense that sequencing reads are processed incrementally in batches, in real-time, as they become available. The displayed results are updated after completion of genus-level analysis of each batch. Detailed analysis is performed “on-demand” in the sense that the user may select or prioritize the genera to be analyzed in detail, so as to focus the laptop’s computational resources on the analysis of specific organisms of interest. The displayed results are updated after completion of the analysis of each batch.

**Fig. 1:**
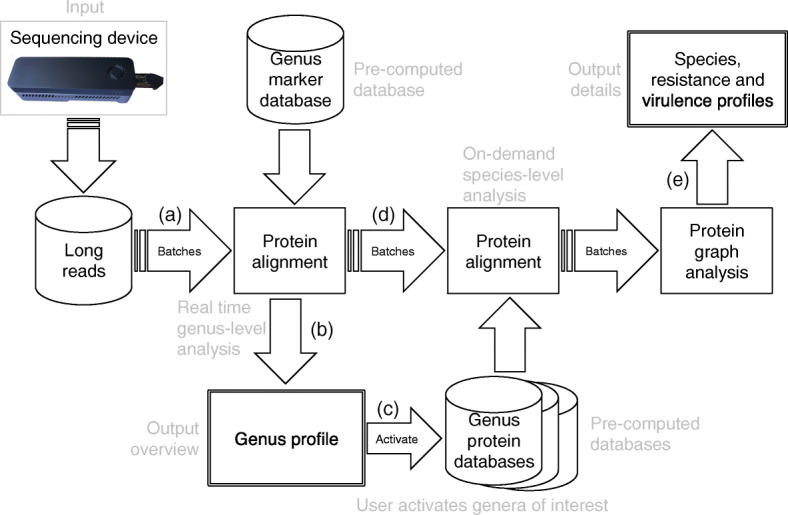
MAIRA workflow. In an online manner, **a** batches of long reads obtained from a sequencing device are subjected to protein alignment against a precomputed database of genus-specific marker genes. Based on this, **b** the list of detected genera is updated in real-time. **c** Substantial evidence for the presence of a specific genus triggers **d** batch-wise protein alignment against a full database of proteins associated with the genus. Based on this, a protein graph is updated and used to perform species and-strain level analysis of the long reads, including the identification of antibiotic resistance and virulence factors

We will now discuss the main workflow of MAIRA, summarized in Fig. 1.

### Input

The input consists of files of sequences in FastA or FastQ format. Input can be specified as a single file or a directory of input files. All reads are then loaded and processed by the program in batches. Alternatively, one can specify a “monitor directory” that the program will periodically inspect to determine whether any new files of reads are available, which are then processed in batches. In particular, when sequencing with a MinION and running a MinIT to perform base-calling, the MinIT can write FastQ files onto its local hard drive, which can be accessed remotely from a laptop and MAIRA will load its input from there.

### Genus-level alignment and analysis

As indicated in Fig. 1a, MAIRA processes incoming reads in batches (by default, 10,000 reads per batch). These reads are aligned against a precomputed database of genus-marker genes, currently representing 2,418 genera, based on the bacterial RefSeq protein database.

Frame-shift aware DNA-to-protein alignment is performed either using LAST [11] or the built-in alignment tool ELLA (manuscript in preparation). For aligning reads against a database both aligners use a frame-shift penalty of 15 and a multiplicity value of 10 (for the marker database) and 50 (for genus-specific databases, see below), respectively. ELLA is written in Java and uses the same algorithmic approach as LAST. It has similar specificity and sensitivity, but is about 2.5 times slower. We have implemented and incorporated ELLA so as to make MAIRA completely self-contained and platform-independent.

The marker database that we provide for download contains 9,434,634 marker genes. All results presented in this paper were obtaining using this database.

The database was precomputed from data downloaded from RefSeq in February 2019, comprising 142,092 bacterial genomes in 2,420 different genera. For each genus, we first constructed a graph whose nodes are proteins and any two nodes *v* and *w* are connected by an edge from *v* to *w*, if the two protein sequences have a similarity of ≥90*%* and if *v* covers at least 90% of *w*, using DIAMOND [12] in BLASTP mode.

A set of candidate marker genes was then computed by greedily determining a minimum *dominating set* of nodes, that is, a subset of nodes *D* such that every node of the graph is either contained in *D*, or is adjacent to some node in *D*. We then turned each candidate set of proteins into a set of genus representative proteins. This was done by aligning all pairs of candidate sets from different genera against each other using DIAMOND, applying an identity threshold of 80% and a coverage threshold of 80%, so as to detect and remove proteins that are not unique to a single genus.

Based on these sets of representative proteins, we then computed the marker database. Using the graph described above, we first removed all nodes that are not associated with a representative protein. Next, we greedily selected a minimum set of nodes so that all genomes are covered by a fixed minimum number of proteins, namely 1000, 500 or 100, for each of the large, medium or small database, respectively. Here, a genome is considered covered by one of its proteins *v*, if *v* is selected, or if *v* is adjacent to a selected node.

This resulted in 9,434,634 genus-specific *marker proteins* that represent 2,418 genera, with a median of 1,121 (mean 3,903) marker proteins (s.d. 10,588). There are 4 genera that contain less than 100 proteins and there are 6 genera that contain more than 100,000 proteins, with Bacillus (205,864), Streptomyces (194,850), and Lactobacillus (158,640) having the largest counts.

While aligning reads against the genus-marker database, for each genus *G*, we maintain and report a *presence scorep* that heuristically seeks to approximate the probability of genus *G* being present in the sample. This is calculated as follows: First, each marker protein *v* associated with *G* is given a *weight*$\omega (v)=\frac {1}{\bar n}$, where $\bar n$ is the average number of marker proteins over all genomes that either contain the marker protein *v* itself or a similar one (both, similarity and coverage ≥90*%*). The presence score for *G* is then given by the sum of weights of all marker proteins for *G* to which a read has been aligned to.

The genus-level analysis of the content of the sample being sequenced is summarized on a tree in the main window of the program. Within minutes, the program will provide a first crude estimation of the genus-level content of the sample being sequenced (or read from an input file). With the completion of genus-level analysis of each batch, this summary is updated.

### Species-level alignment

The above described genus-level analysis is designed to provide a very quick taxonomic analysis of long read sequencing data, akin to traditional 16S rRNA taxonomic profiling.

In addition, the program is able to perform an in-depth, protein-alignment-based taxonomic and functional analysis of the incoming reads, in batches. To save computational resources, the in-depth analysis is performed on demand, for a specified subset of taxa.

In more detail, using the bacterial RefSeq protein database, for each genus we pooled all proteins that either correspond to the genus itself, or to an ancestor or descendant taxon. We obtained a set of 2,420 genus-specific databases, each containing an average of 43,849 proteins (median 6,310, s.d. 251,520).

Batches of incoming reads (Fig. 1d) are aligned against the reference databases for all genera that have been “activated” (Fig. 1c), either explicitly by the user, or automatically, because their presence score has exceeded the activation threshold (80%, by default). The program tracks all batches of reads and ensures that each batch of reads will eventually be compared against every activated genus-specific database, independent of when the batch of reads was loaded into the program or when the genus was activated.

### Protein synteny graph

The alignments computed during species-level analysis are used to build a *protein synteny graphPSG*. In this graph, each node represents a protein gene family. Gene families are defined as follows: The alignments produced from the genus specific databases for each long-read are pooled together, and filtered by the coverage of the reference protein (default: 80%), the percentage of positives in the alignments (default: 60%), and the raw-score of the alignment (default: 100). The alignments that stack (at least 2/3 of the smaller alignment overlapping with the longer alignment) at different loci are then binned together so as to obtain gene families. These bins are represented as *protein nodes* in the graph. Each such *protein nodev* is annotated by the set *τ*(*v*) of all taxa and functional classes associated with members of the gene family.

A protein node is placed into the graph, if there exists a sequencing read that aligns to a gene sequence in the corresponding family. Two such nodes *v* and *w* in the graph are joined by an undirected edge {*v*,*w*} if the two corresponding gene families appear next to each other in some sequencing read. Each edge *e* is annotated by the set *ρ*(*e*) of all such reads.

Let *t* be a taxon. The taxon-specific *induced* protein synteny graph *PSG*|_*t*_ is given by the set of all protein nodes *v* whose annotation contains the taxon *t*, that is, for which *t*∈*τ*(*v*) holds, together with all other nodes that lie between them in the graph along some read. Any two nodes in the resulting graph are connected by an edge, if and only if they were in the original graph.

### Taxonomic analysis

Using these concepts, we declare a taxon *t* to be *present* in the sample, if the corresponding induced graph *PSG*|_*t*_ contains enough nodes. In more detail, we define the naïve *completeness score* *k*(*t*) as the number of nodes in the induced graph divided by the median number of proteins present for the same or (a similar) taxon in the RefSeq database. A taxon is considered present, if its completeness score exceeds a specified threshold (80%, by default).

We further compare all induced graphs to all others that are above the detection criteria, and eliminate those that are largely contained within the induced graph of another taxon (default: 85% of the nodes), without containing the same percentage of the nodes from the other itself. We do this in order to eliminate false-positive taxa that can still produce highly connected and complete subgraphs due to partial but not complete similarity to the true positive taxon.

### Antibiotic resistance and virulence analysis

To identify potential antibiotic resistance genes, we use information from the *comprehensive antibiotic resistance database* (CARD) [4] to annotate nodes that represent resistance-associated genes. Similarly, to identify potential virulence factors, we use information from the *virulence factor database* (VFDB) [5] to annotate nodes that are considered virulence factors. For a given taxon *t*, we report all CARD and VFDB annotations present in the induced graph *PSG*|_*t*_.

## Results

We demonstrate the use of the program using three datasets.

### Nanopore mock community

The Nanopore mock community published in [14] consists of 3,491,078 long reads with an average length of 4,012 bases, sequenced on a GridION. The source consists of eight bacterial strains and two yeast strains.

**Fig. 2:**
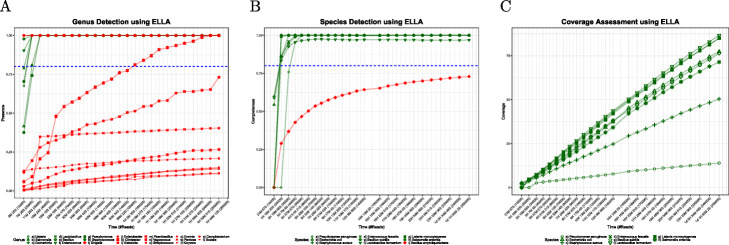
Performance on mock community. **a** The presence score for true positive (green) and false positive (red) genera as a function of computation time and number of reads processed. The blue line indicates the threshold used to deem a genus “present”. **b** The completeness of true positive (green) and false positive (red) species as a function of computation time and number of reads processed. The blue line indicates the threshold used to deem a species “present”. **c** The coverage of species, as a function of time and number of reads processed

We ran our pipeline on 25 batches of 10,000 reads each, using both LAST and ELLA. In both cases, MAIRA correctly determined all 8 present genera of bacteria after the third batch (requiring 5 minutes using LAST and 11 minutes using ELLA). Both aligners also resulted in false-positive identification of 2 genera after the third batch: *Enterobacter* and *Shigella*. Another false-positive *Citrobacter* also passed the default genus detection threshold of 0.8 for the 15-th batch for ELLA and 11-th batch for LAST (see Fig. 2a).

Running the species-level analysis to completion on 25 batches required $9\frac {1}{2}$ hours using LAST and nearly 26 hours using ELLA. All eight true-positive species were identified with strong signal (completeness >90%) using both aligners after the end of the 4-th batch, after one hour for LAST and three hours for ELLA; and their scores thereafter remained constant.

MAIRA identified one false-positive species, *Bacillus amyloliquefaciens*, using both aligners. Its completeness score, however, stayed below 0.75 using ELLA and 0.85 using LAST (see Fig. 2b). We report the coverage of genomes in Fig. 2c.

Running the software on further batches eventually produces one more false positive genus *Klebsiella*.

### Simulated pathogen mock community

We now consider the simulation of a pathogen mock community. We simulated an even community consisting of ten different bacterial species, each represented by 10,000 simulated Nanopore reads generated using NanoSim [15]. The ten source genomes correspond to the components of a commercially available metagenomic mock community for pathogen detection called ATCC^Ⓡ^ MSA-4000^TM^. This consists of 11 strains of 10 species in eight different genera. The genome sequence of one of the strains (ATCC BAA-1718) is very fragmented (174 contigs) and so we excluded it from our study.

Complete analysis of all 100,000 simulated long reads took slightly over six hours using MAIRA and LAST. The approach correctly determined all eight genera at the end of the 7-th batch, while indicating two incorrect genera, *Shigella* and *Enterobacter*. The species-level analysis resulted in identification of all 10 species present, while indicating three incorrect species, *Staphylococcus mitis*, *Streptococcus pseudopneumoniae* and *Streptococcus oralis*. Note that the dataset contains multiple strains of both *Staphylococcus* and *Streptococcus*.

To assess the ability of MAIRA to correctly determine the presence of CARD or VFDB genes, for both functional classifications, we aligned the set of their reference proteins against all coding sequences (CDSs) in the source genomes, using DIAMOND [12]. Then, for both classifications, any protein that aligned to a CDS with a percent positives of ≥80% and a reference coverage above ≥90% was considered an actual positive.

For each species, MAIRA recovered almost all actual positives for both CARD and VFDB, with mostly low numbers of false positives, see Table 1.

**Table 1:** Assessment of CARD and VFDB hits reported by MAIRA

Organism	Classif.	TP (CDS)	FN (CDS)	TP (Term)	FP (Term)	FN (Term)
*Acinetobacter baumannii*	CARD	25	3	145	6	65
	VFDB	31	9	31	0	10
*Enterococcus faecalis*	CARD	22	5	24	2	26
	VFDB	23	3	23	0	5
*Escherichia coli*	CARD	69	6	81	22	22
	VFDB	102	27	123	0	36
*Klebsiella pneumoniae*	CARD	63	7	85	20	282*
	VFDB	33	8	34	0	8
*Neisseria meningitidis*	CARD	15	1	16	1	5
	VFDB	31	8	30	0	12
*Pseudomonas aeruginosa*	CARD	55	15	87	7	38
	VFDB	119	16	119	0	22
*Staphylococcus aureus*	CARD	27	2	32	2	7
	VFDB	57	5	55	0	5
*Streptococcus agalactiae*	CARD	4	7	4	4	20
	VFDB	31	0	32	0	1
*Streptococcus pneumoniae*	CARD	17	1	19	8	14
	VFDB	11	2	11	0	4
*Streptococcus pyogenes*	CARD	4	8	4	4	16
	VFDB	29	1	29	0	3

For all ten bacterial species present in a simulated pathogen mock community, we report the number of true postives (TP), false positives (FP) and false negatives (FN), based on the coding sequences (CDS) in the source genomes, and based on the terms reported (Term). (^∗^) The large false negative value for CARD terms reported for *Klebsiella pneumoniae* is based on a single CDS that is annotated with over 250 different CARD terms (WP_004176269.1) and thus gives an exaggerated impression

### Carbapenemase-producing gram-negative bacteria isolates

The long read dataset published in [16] consists of 110 MinION long read sequencing datasets, of which we were able to download 109 (sample ERR2797062 could not be found). These datasets were sequenced from 58 *Klebsiella pneumoniae*, 28 *Escherichia coli*, 13 *Pseudomonas aeruginosa*, and 10 *Acinetobacter baumannii* isolates. The authors report that 63 isolates are suspected to have carbapenemase resistance, 34 have other types of antibiotic resistance, and indicate that they failed to produce evidence for antibiotic resistance in 13 isolates.

We ran MAIRA on the first 10,000 reads of each sample (or all reads, where there were less than 10,000). It took about one minute per sample to complete genus-level analysis using MAIRA and LAST.

For all 109 samples, MAIRA reports the correct species and no false positives. However, the initial, fast genus-level analysis did report some false positive genera. For example, in the case of *Klebsiella pneumoniae*, three false positives were listed. None of the false positive genera were confirmed by the species-level analysis. The full running time varied between five and 20 minutes per sample, depending on the organism.

In order to determine carpabanem-resistance of isolates, we checked for CARD ontology terms with annotation *confers_resistance_to: ARO:0000020* and found homology-based evidence of resistance in 102 isolates with varying ranges of support. The number of reads covering resistance genes ranged from 1 to 35.

## Discussion

**Fig. 3:**
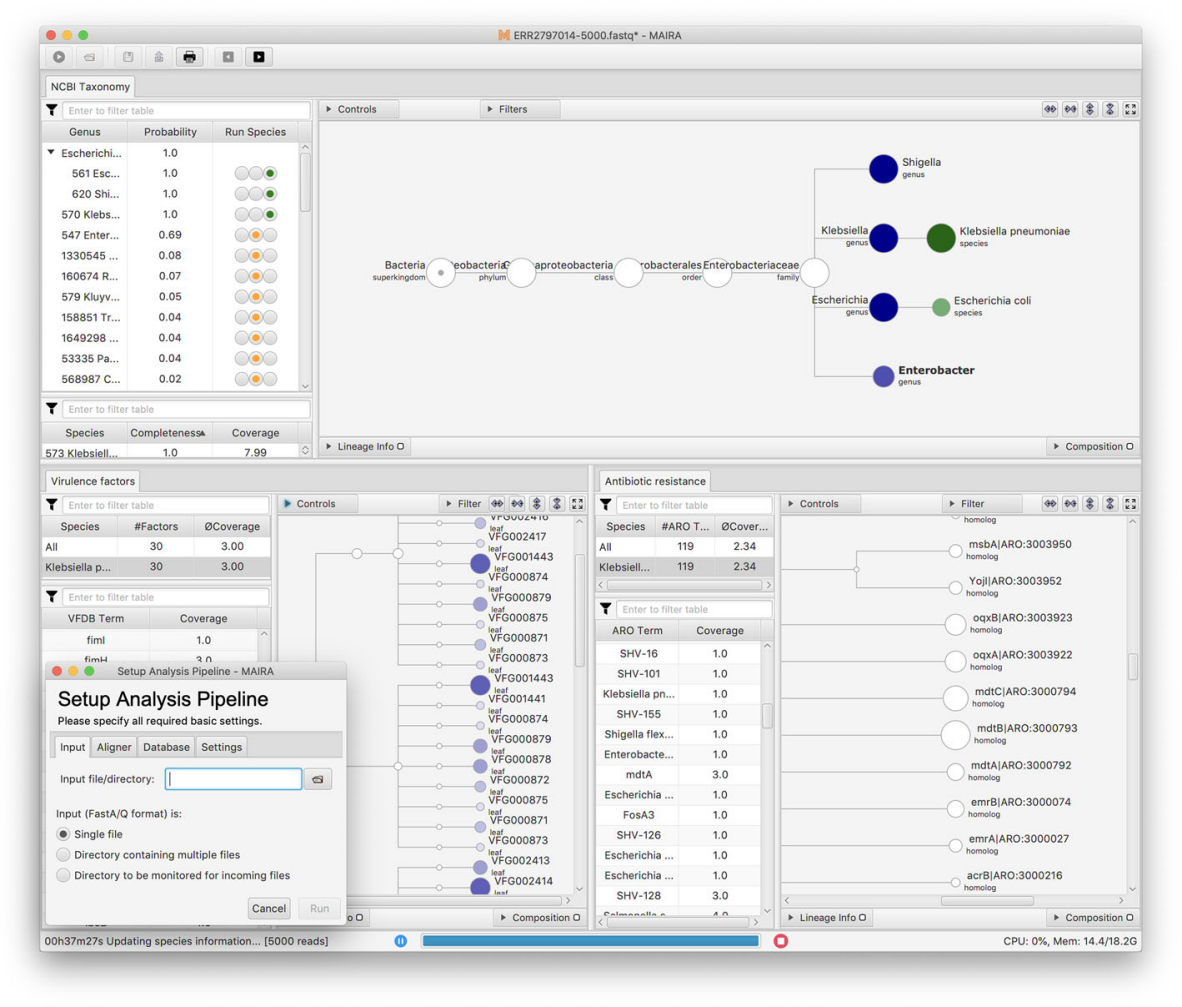
MAIRA user interface. During processing of an input file or directory, MAIRA provides an overview of the genera, species and functional classes discovered so far. The user can explore and download results while they are being produced, so as to allow insights into the processed sequences

In Fig. 3 we show a screen shot of the user interface. The user sets up an analysis using the dialog shown at the bottom left. The program then processes the given data in batches, frequently updating the tables and trees that represent the taxonomic and functional analysis of the data. The user can interactively explore the taxonomic and functional analyses (CARD and VFDB), can export all tables and trees in different formats, save and reopen the analysis.

With MAIRA, we intend to provide a powerful and accurate, standalone long read analysis software that runs on a laptop and can be used in the field. The use of a comprehensive protein reference database ensures wide applicability of the software. The two step approach, light-weight genus-level analysis, followed by on-demand species-level analysis of selected genera, allows the user to interactively control how the computational resources of the laptop are used.

The presented results suggest that the software is able to perform analysis of real data in acceptable running time and with very good accuracy. Alternative approaches currently rely on the use of servers or cloud resources to perform detailed analysis.

MAIRA is a general purpose tool. Moving forward, we intend to add special purpose modules that are dedicated to specific applications, such as the detection of specific pathogens. Moreover, antibiotic resistance is a complex question that is not adequately addressed by the tool at present. In future work, we intend to also consider DNA alignments of resistance-related genes to perform a more detailed analysis. Future versions of the program will use locality of resistance genes and virulence factors in the protein synteny graph to provide more meaningful results.

Initial genus-level analysis performed by MAIRA is very fast and can be applied online during sequencing. Detailed, species-level analysis on individual genera is also fast enough to keep pace with a current MinION+MinIT sequencing setup. Complete analysis of one Gb of long read data requires about 10 hours using LAST and can be considered comfortably doable on a laptop.

One current limitation is that MAIRA focuses on identifying genera and species. Moving forward, we plan to lift this restriction so that assignments can be made to higher level taxonomic ranks, as well. Another limitation is given by the choice of reference databases. In this paper we describe the use of bacterial RefSeq, and the use of this database will avoid false positive identifications. If false negative results are to be avoided, then the use of the more comprehensive, but less curated, NR database may be more appropriate.

The data analysis presented here suggests the current algorithm for performing a fast genus-level analysis tends to yield false positive genera that are then not subsequently confirmed by the detailed species-level analysis. This indicates that there is room for improving the accuracy of the fast genus-level analysis and we will address this in future work.

### Comparison with centrifuge

In order to further evaluate the performance of MAIRA, we compared it to Centrifuge [17], which is the backend for the real-time analysis software WIMP [18], developed by ONT. Centrifuge is designed to work with nucleotide databases on a server. We designed our evaluation so as to simulate a common obstacle in metagenomics, namely that the organism to be identified is not present in the reference databases. For this, we extracted genome assemblies from RefSeq that were published before 2016, and built MAIRA databases for them, as explained above. For Centrifuge, we were only able to build an index for the subset of complete assemblies, as using all assemblies produced an out-of-memory error on a server with 600GB of RAM. Also, the pre-built NCBI-nt index obtained from the Centrifuge website produced the same error.

We used NanoSim to collect 20x long reads on each of 100 different genomes available after 2016. These genomes were selected so as to equally cover four different categories, reflecting what information is available for the corresponding MAIRA and/or Centrifuge indices.

The 4 categories are: (a) no information at species level for MAIRA or Centrifuge; (b) no information at species level for Centrifuge, some for information for MAIRA from incomplete assemblies; (c) only one genome from the species for both MAIRA and Centrifuge; and (d) more than 15 genomes from the species for both MAIRA and Centrifuge. Species assignment accuracy was based on the top 10% of the scores of each tool (top 10% completeness for MAIRA, top 10% number of reads assigned for Centrifuge).

For category (a), MAIRA reported a false species in 5 cases, and did not report any assignment in the other 20; whereas Centrifuge reported a false assignment for all 25 genomes. For category (b), MAIRA reported the true assignment in 22 cases, failed to assign a species in 3 cases, and reported an additional species in 5 cases; whereas Centrifuge again reported a false assignment in all 25 cases. For category (c), MAIRA failed to identify the species in 3 cases, while Centrifuge failed in one case. MAIRA reported 4 false positives, while Centrifuge reported one false positive. For category (d), Centrifuge reported only the true positive species in all cases, whereas MAIRA failed to identify 4, and reported a false positive for one case.

In summary, Centrifuge shows better performance in the ideal case that the sequenced genomes are present in the reference DNA database. However, in the more realistic scenario in which the sequenced genomes are not present in verbatim in the reference databases, MAIRA shows better performance. Moreover, an added benefit of MAIRA is that it also provides a functional analysis, which Centrifuge does not attempt.

## Conclusions

This paper demonstrates that laptop-based analysis of sequencing reads from mobile sequencing devices is possible and this should extend the range of mobile sequencing. As an easy-to-install, standalone application that runs on all three major operating systems, MAIRA provides a complete analysis solution that does not require access to additional computational resources.

Beyond the practical uses of the software, this work illustrates that protein-alignment-based analysis can be performed in real-time on a laptop, that an on-demand design allows a user to direct their computational resources to species of interest, and it shows that the protein synteny graph is a useful concept for the analysis of long reads. Moreover, while designed for running on a laptop, the command-line version can also be run on a server, where the user can also benefit from its efficient design.

We believe that this type of software may play an important role in practical pathogen detection in the future [19].

## Data Availability

The publicly available datasets used in the study are available from following BioProject accessions: PRJEB29504 and PRJEB28660. Project name: MAIRA - mobile analysis of long reads. Project home page: https://ab.inf.uni-tuebingen.de/software/maira Operating system(s): Platform independent. Programming language: Java (OpenJDK 12 and OpenJFX). Other requirements: High-end laptop, ≥32 Gb of memory, 500G of SSD License: GNU GPL Any restrictions to use by non-academics: None

## Abbreviations

PSG: protein synteny graph
CDS: coding sequence
Gb: gigabase
